# From Simulation to Healthcare: KINAITICS' AI Framework for Cyber-Physical Security

**DOI:** 10.12688/openreseurope.21717.1

**Published:** 2026-05-15

**Authors:** Paolo Marcheschi, Maria Pisani, Stefano Dalmiani

**Affiliations:** 1 BioInformatica Traslazionale ed eHealth (BITE), Fondazione Toscana Gabriele Monasterio per la Ricerca Medica e di Sanita Pubblica, Pisa, Tuscany, 56011, Italy

**Keywords:** Artificial Intelligence (AI), Cyber-Physical Systems (CPS), Cybersecurity, Electronic Health Records (EHR), Social engineering Bot detection, Behavioral analysis, Human-in-the-loop (HITL), Explainable AI (XAI), Threat matrix, Data scraping, Critical infrastructure Structural Health Monitoring (SHM)

## Abstract

The increasing integration of Artificial Intelligence (AI) into Cyber-Physical Systems (CPS) presents complex cybersecurity challenges, necessitating a reevaluation of traditional threat assessment. The KINAITICS project addresses these evolving threats by conducting in-depth research into cyber-kinetic attacks, where malicious cyber activities manifest as real-world physical disruptions. The project is also dedicated to developing resilient, AI-driven defense mechanisms.

This paper outlines KINAITICS’ foundational work, including the creation of a tailored KINAITICS Threat Matrix (KTM). This innovative framework systematically identifies, categorizes, and assesses threats unique to AI-integrated CPS. The paper details the KTM’s practical application across five high-stakes use cases, ranging from safeguarding nuclear facility simulations to protecting electronic health record (EHR) systems from sophisticated phishing attacks.

A central focus of the KINAITICS project is the rigorous development and evaluation of both offensive and defensive AI tools. These tools are designed to investigate, understand, and mitigate the multifaceted threats posed by cyber-kinetic adversaries. The overarching objective is to significantly enhance the resilience of critical infrastructures against advanced cyber-physical threats, ensuring the continued safety, security, and operational integrity of systems vital to modern society.

## Introduction

### Cyber-kinetic attacks using Artificial Intelligence

The pervasive use of AI in Cyber-Physical Systems (CPS) generates critical new cybersecurity risks, necessitating a thorough redefinition of threat and risk assessments to address the dynamic interaction between the cyber and physical worlds. The core ambition is to conduct in-depth technical research to understand these emerging threats and develop resilient defense approaches.

**Figure 1.**
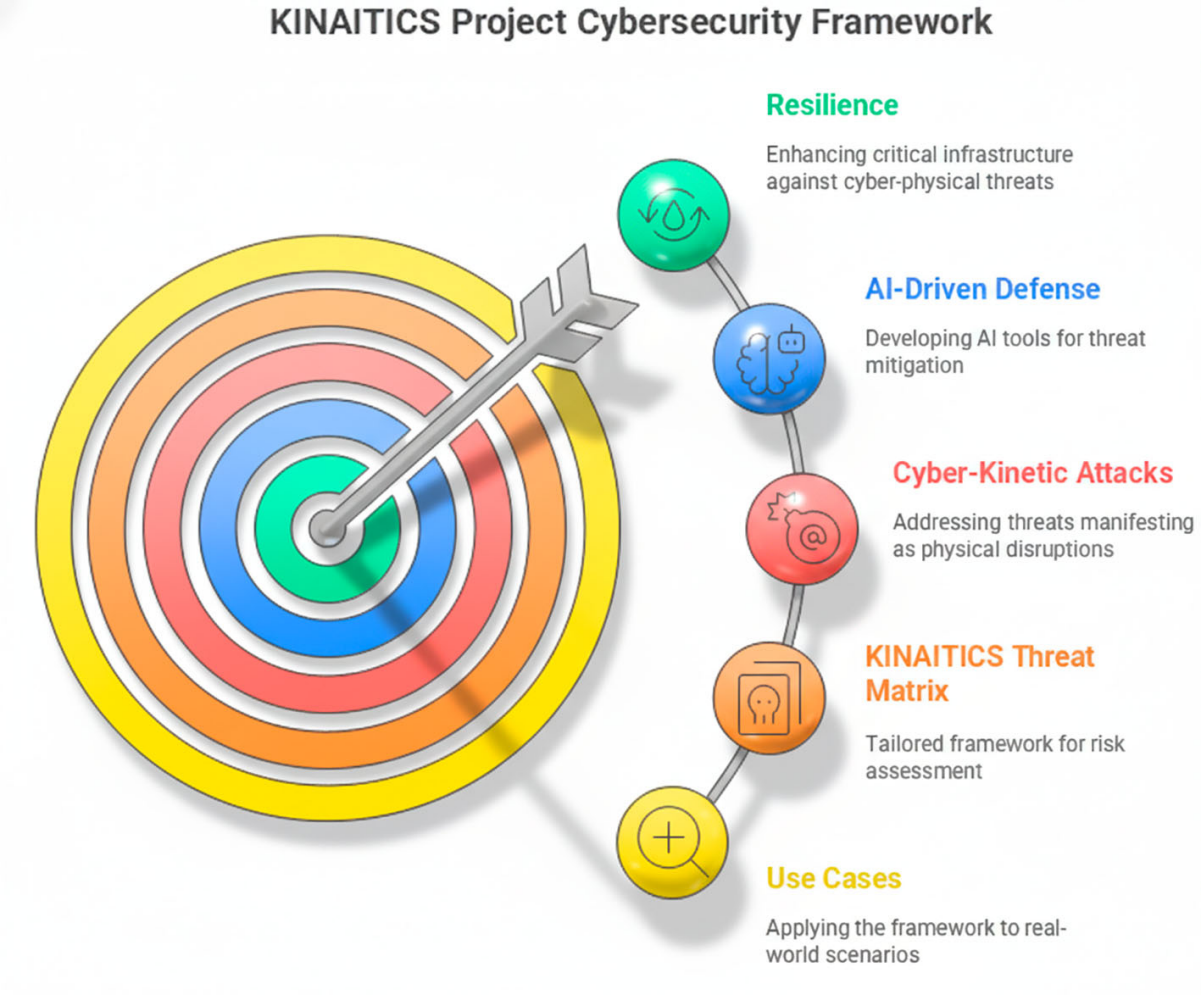
Kinaitics project cybersecurity framework.

**Figure 2.**
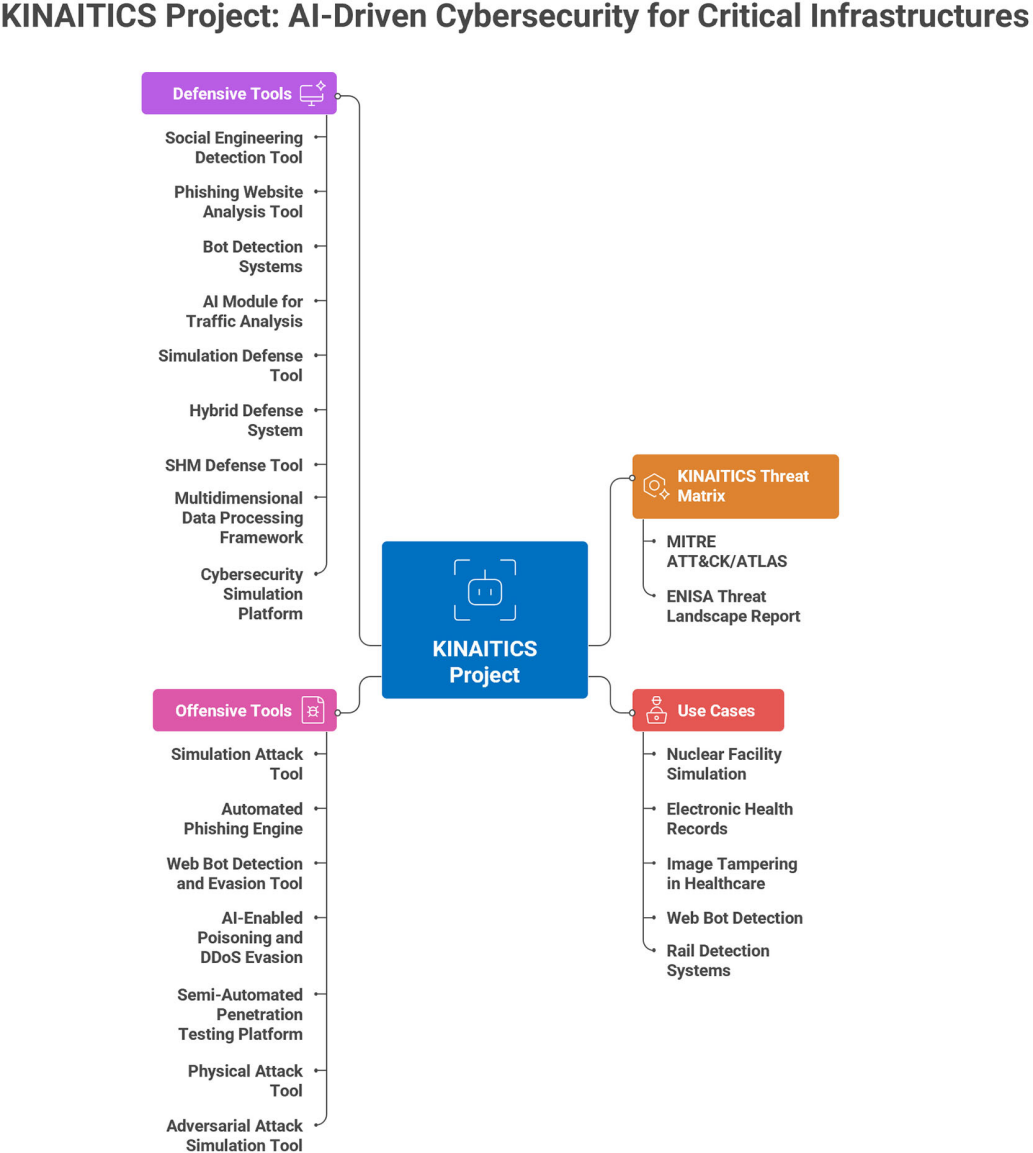
Kinaitics architecture.

**Figure 3.**
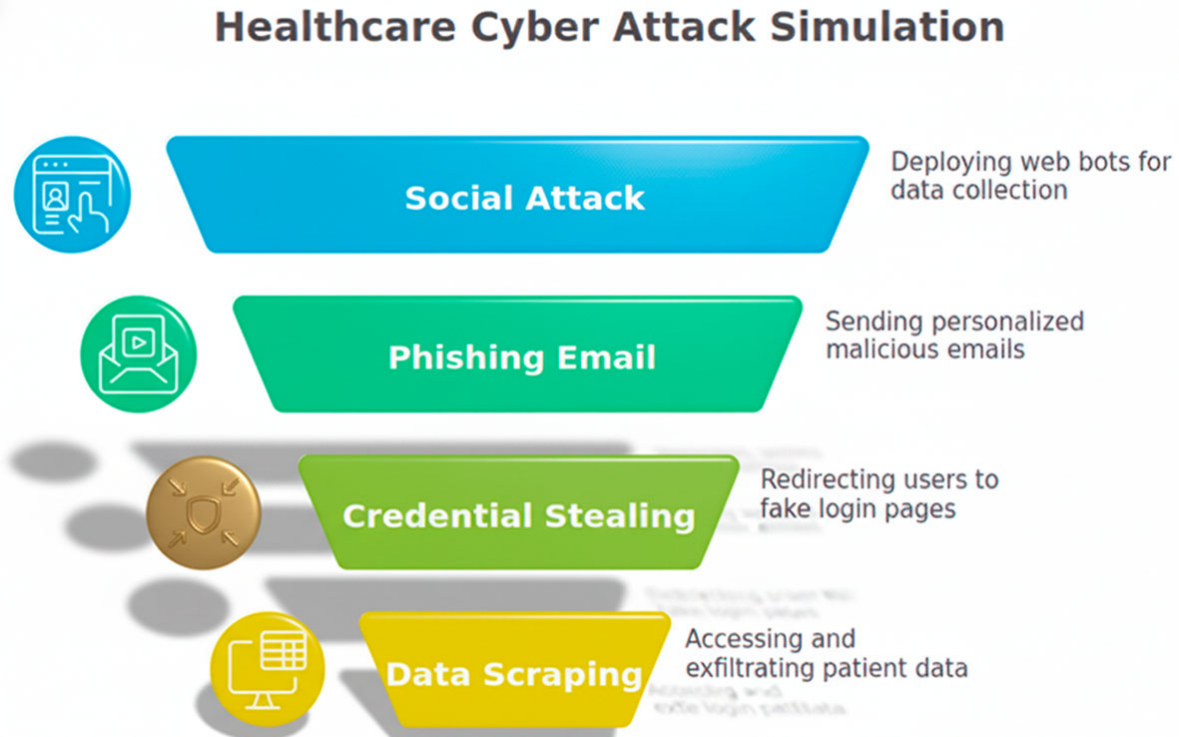
Attack scenario process.

**Figure 4.**
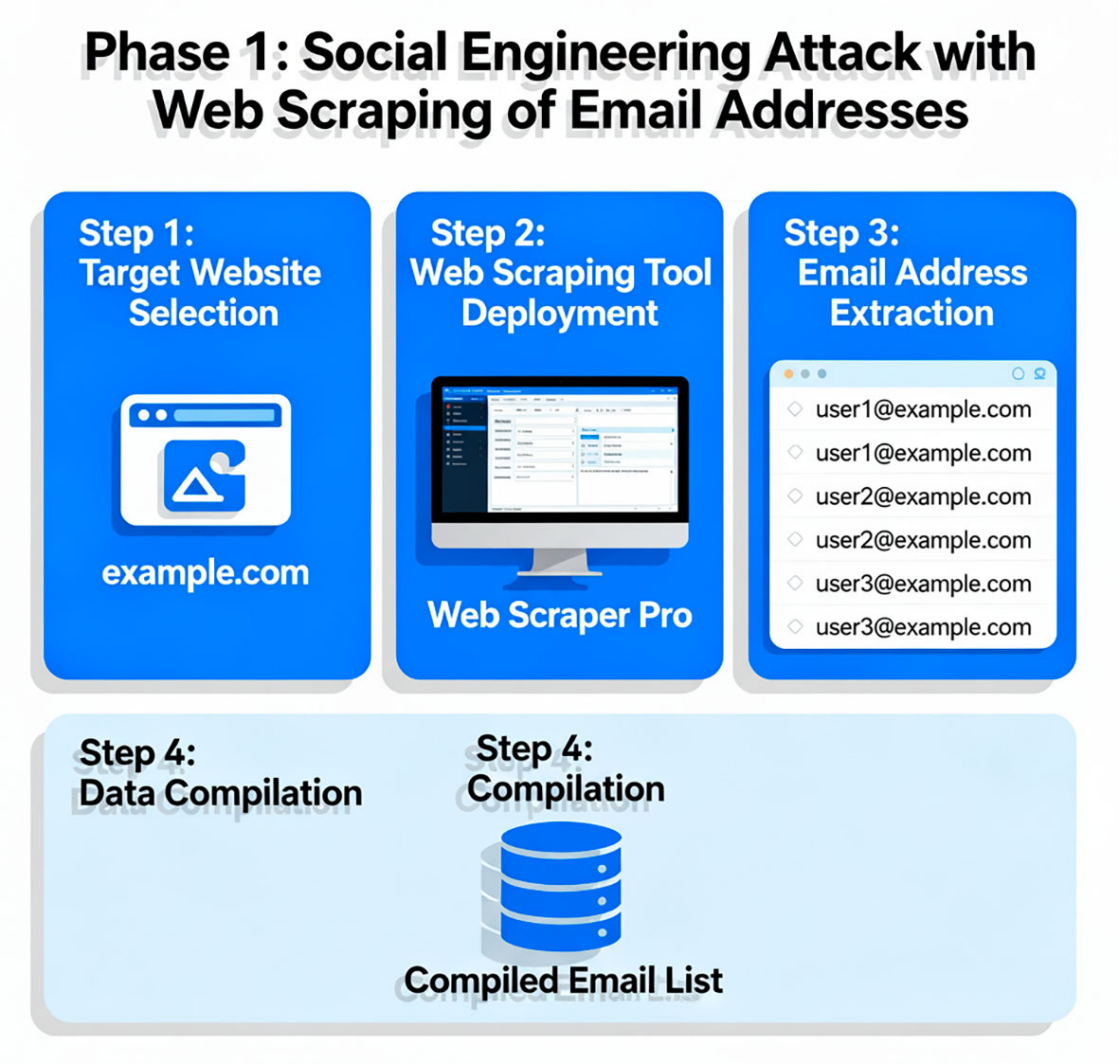
Social attack.

**Figure 5.**
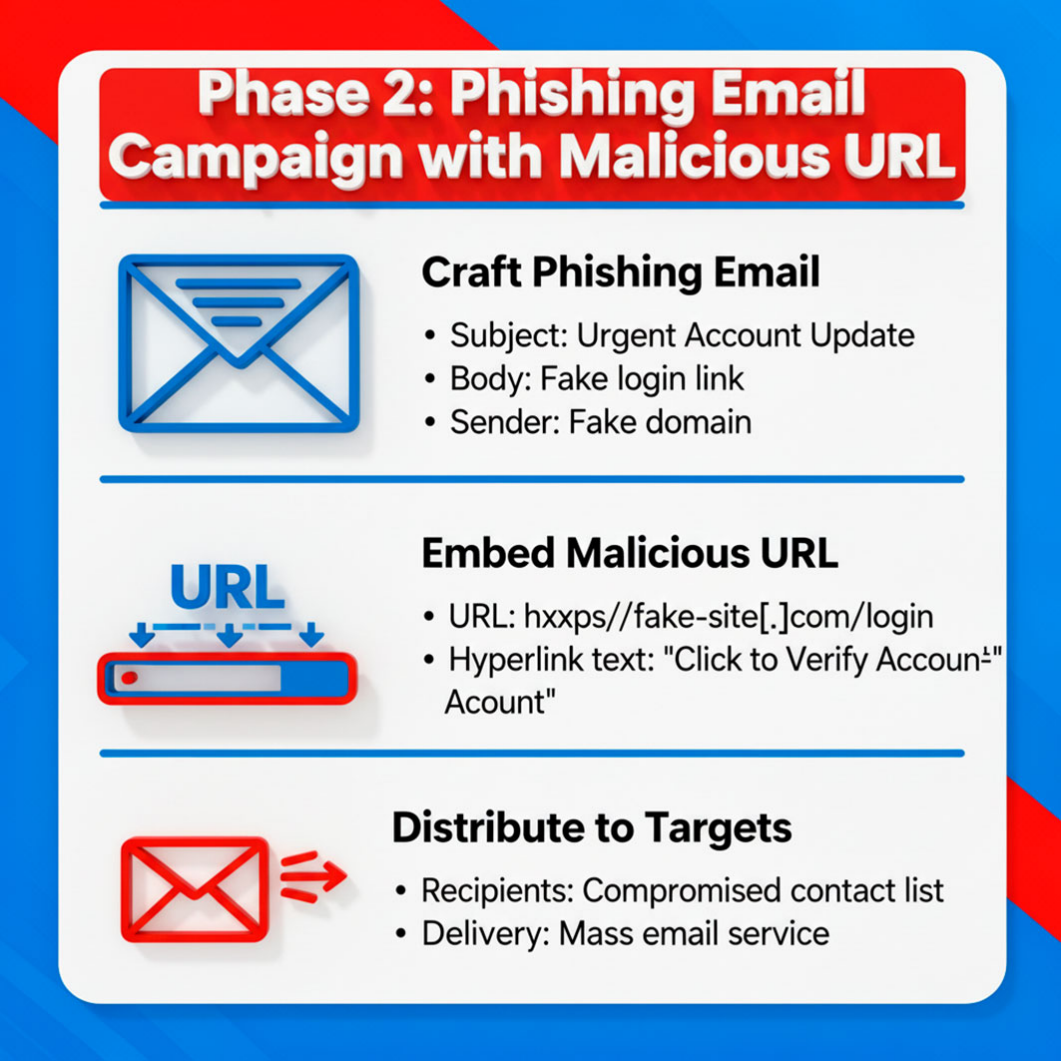
Email phishing.

**Figure 6.**
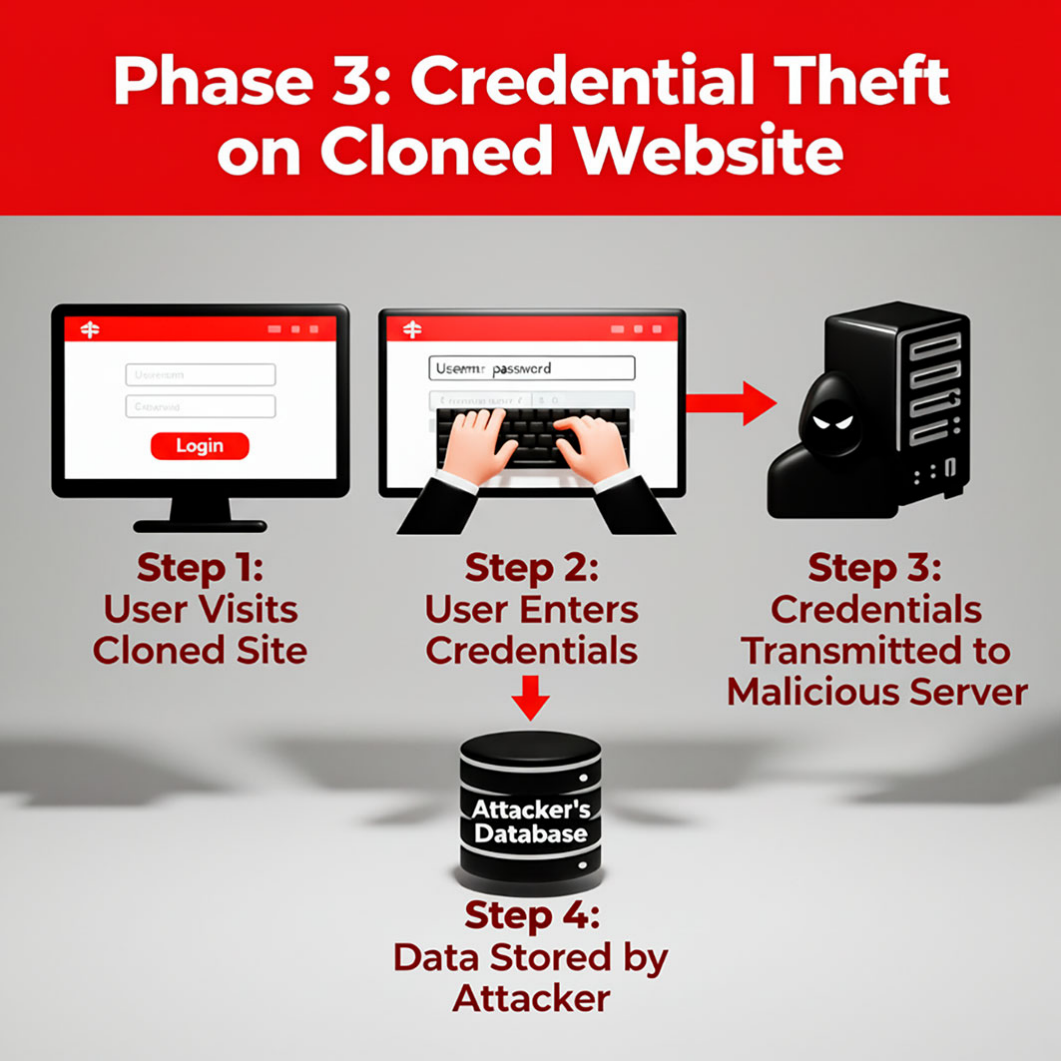
Credential stealing.

**Figure 7.**
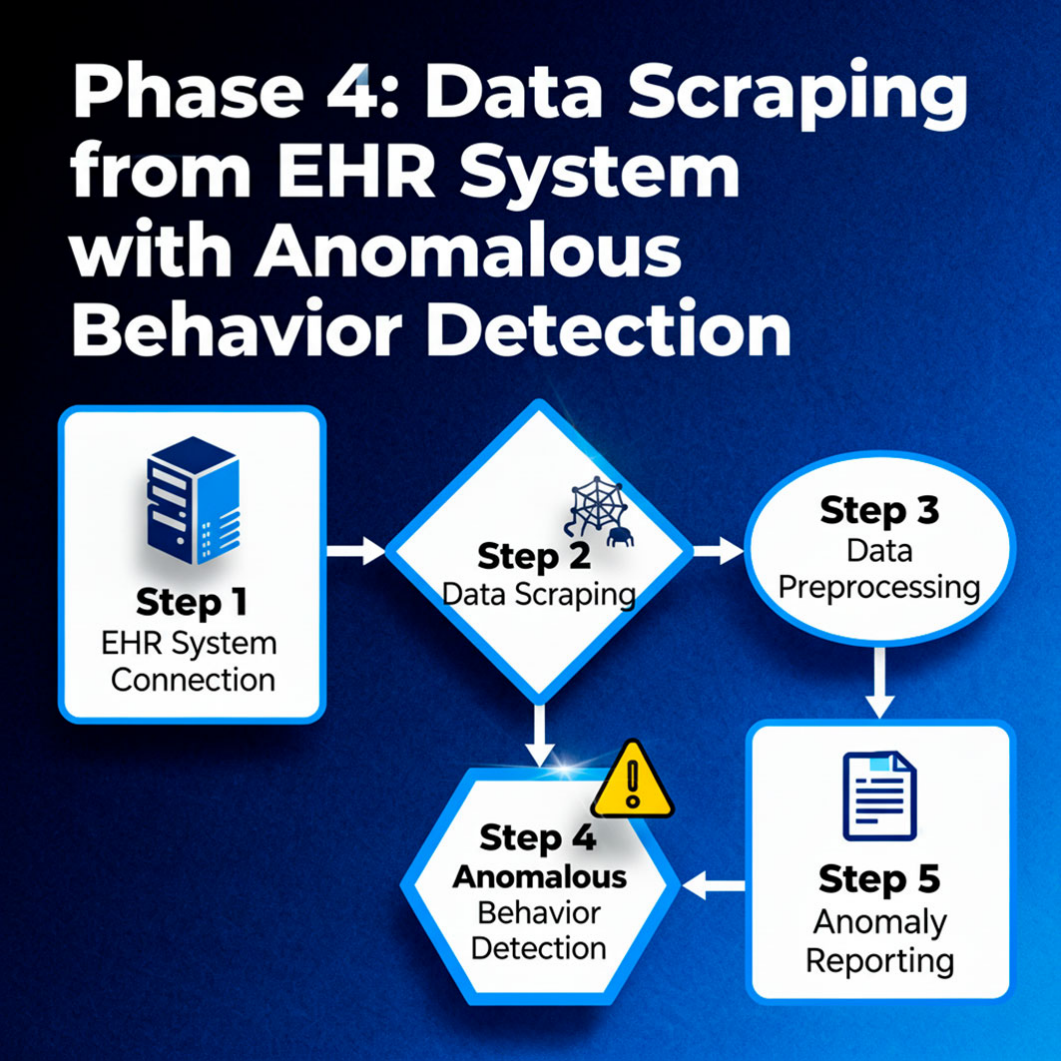
Data scraping.

**Figure 8.**
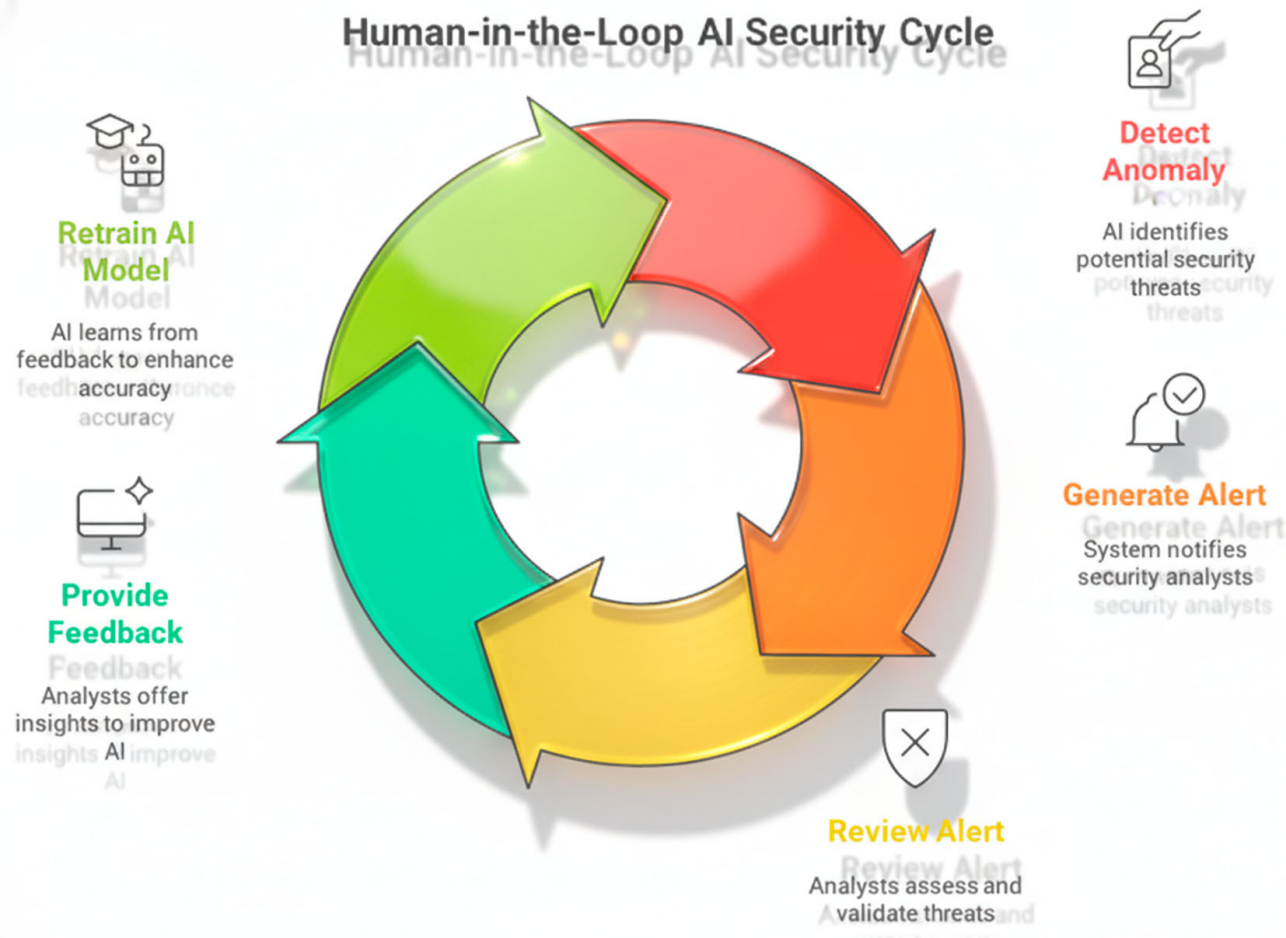
Human-in-the-Loop AI security cycle.

**Figure 9.**
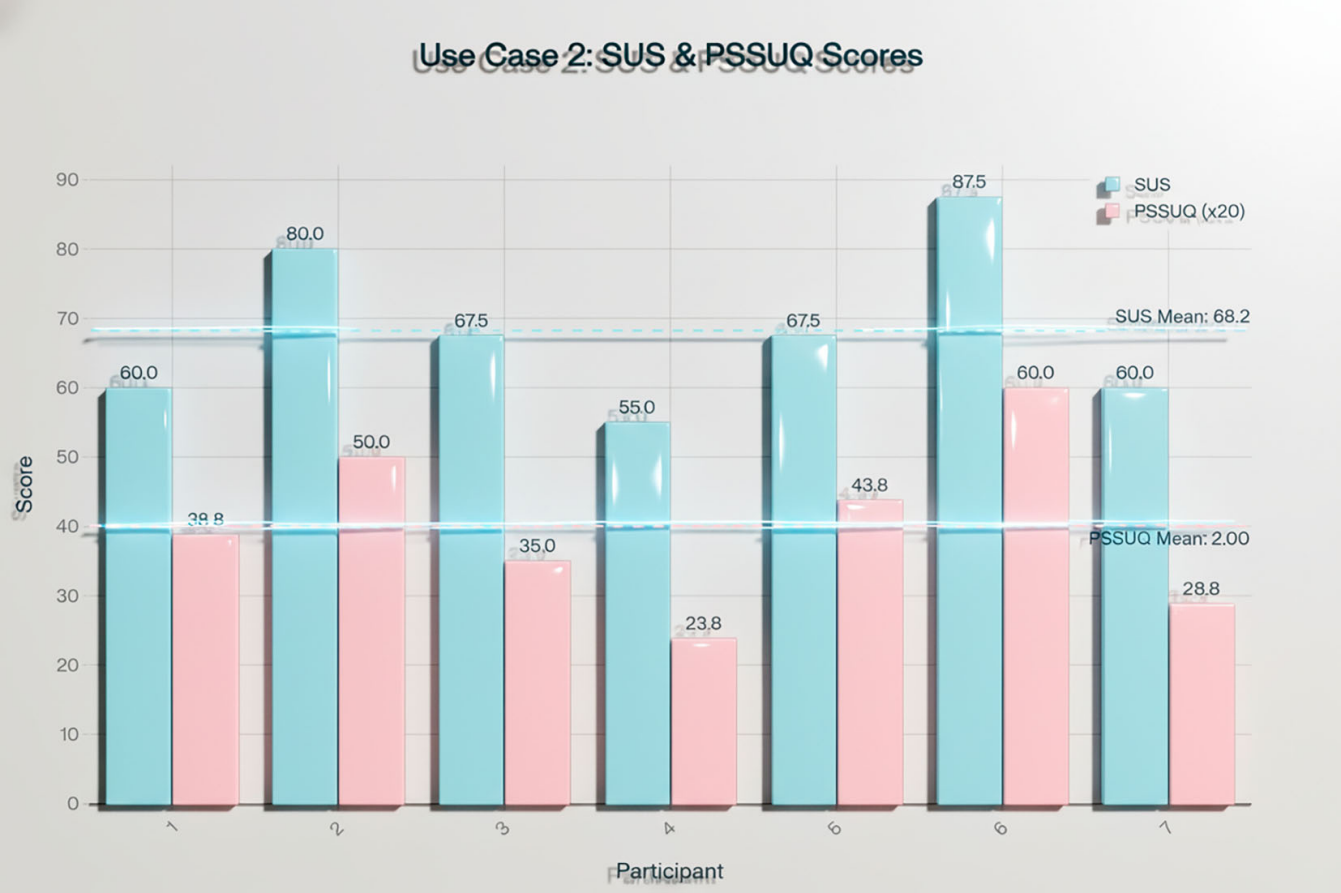
Use Case 2 SUS and PSSUQ scores.

**Figure 10.**
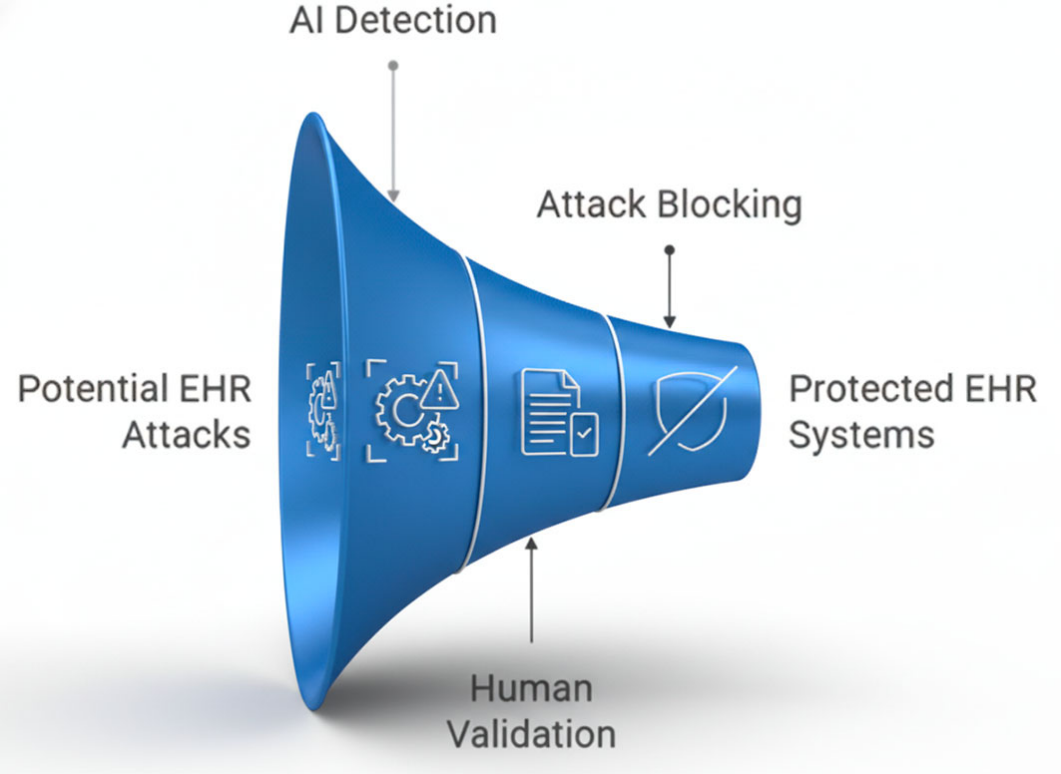
EHR defence strategy funnel.

**Table 1.** Kinaitics use cases.

Use case	Core sector/focus	Attack vector/scenario	Key defense mechanism & result
**UC1**	**Energy (Nuclear Simulation)**: Safeguarding simulation codes for design integrity.	Manipulating input data (input-based attacks) and intellectual property exfiltration (code theft).	The defense mechanisms achieved a **high success rate (over 90%)** in detecting and blocking malicious data points. Forensic and behavioral analysis tools prevented IP exfiltration attempts.
**UC2**	**Healthcare (EHR)**: Defending Electronic Health Record data from phishing and scraping attacks.	Multi-stage attack chain: Social attack, Phishing email, Credential stealing, Data scraping (using valid credentials).	Layered defense combining Social Engineering Detection, Phishing Website Analysis, Bot Detection Systems, and **Human-in-the-Loop (HITL)** validation proved instrumental in mitigating multi-stage attacks.
**UC3**	**Healthcare (Diagnostic Imaging)**: Countering adversarial attacks (tampered images) on AI diagnostic models.	Model poisoning (during training) and inference-time evasion attacks (subtly altering input data).	**Hybrid Defense System (HDS)** detects tampered images/models. Crucially, the incorporation of **human radiologists** ensured verification of AI predictions and identification of imperceptible perturbations.
**UC4**	**Digital Industry (Web Bots)**: Detecting advanced web bots for DDoS and scraping.	Realistic bot traffic and adversarial evasion techniques (FGSM, BIM, PGD).	**Adaptive detection models** (RL-trained via CyberShield) showed remarkable capability to distinguish traffic with a low false positive rate of **0.795%**.
**UC5**	**Critical Infrastructure (Structural Health Monitoring, SHM)**: Securing AI-based rail defect detection systems.	Backdoor attacks, adversarial attacks, intrusion/hack of the central server, and physical attacks.	Countermeasures like **adversarial training** and **prediction consistency checks** ^23, 25^ proved highly effective in improving model robustness and ensuring continuous and reliable operation.

**Table 2.** Use Case 2 - Sus PSSUQ table.

Metric	Score	Standard deviation	Range	Interpretation
**SUS Overall**	**68.2**	11.70	55.0–87.5	**Good usability**
**Individual SUS Scores**	**[60.0, 80.0, 67.5, 55.0, 67.5, 87.5, 60.0]**			
**PSSUQ Overall**	**2.00**	0.62	1.00–7.00	**Above average usability**
**SYSUSE (System Usefulness)**	**1.60**	-	1.00–7.00	System utility and ease of use
**INFOQUAL (Information Quality)**	**2.64**	-	1.00–7.00	Quality of system information
**INTERQUAL (Interface Quality)**	**1.67**	-	1.00–7.00	Interface design quality
**Individual PSSUQ Overall**	**[1.94, 2.50, 1.75, 1.19, 2.19, 3.00, 1.44]**			

The KINAITICS project, Figure 1, focuses on addressing cybersecurity risks introduced by the integration of Artificial Intelligence (AI) into Cyber-Physical Systems (CPS). ^1, 2^ Its core ambition is to conduct in-depth technical research into cyber-kinetic attacks—where cyber threats manifest as physical disruptions—and to develop resilient AI-driven defense mechanisms. The project has established a tailored KINAITICS Threat Matrix (KTM) ^3, 4, 5^ and applies its findings across five high-stakes use cases. These use cases range from safeguarding nuclear facility simulations to protecting electronic health record (EHR) systems from sophisticated phishing attacks, ^6, 7^ image tampering in healthcare, web bot detection, and cyber-physical security for rail detection systems. Standardization is essential for achieving true interoperability and advancing security strategies. This requires the rigorous adoption of established and widely accepted protocols for health information exchange. Key among these are HL7 (Health Level Seven) ^8, 9, 10^ a set of international standards for transfer of clinical and administrative data between software applications used by various healthcare providers, which includes versions like HL7 v2, v3, and the more modern FHIR (Fast Healthcare Interoperability Resources). The integration of such protocols ensures secure, seamless data exchange across disparate Electronic Health Record (EHR) systems and other clinical applications. Equally critical is the adoption of DICOM (Digital Imaging and Communication in Medicine), the international standard for handling, storing, printing, and transmitting information in medical imaging. DICOM defines the format for medical images and their associated data (such as patient demographics, study information, and imaging parameters), and the communication protocols for transferring them, ensuring that images—from X-rays to MRIs and CT scans—can be reliably shared and viewed across different devices and institutions, ^11, 12, 13^ A key aspect of KINAITICS is the development and evaluation of both offensive and defensive AI tools to investigate and mitigate these complex threats, ultimately enhancing the resilience of critical infrastructures against advanced cyber-physical adversaries.

## Method

The project meticulously examined a diverse range of critical applications to bolster cybersecurity and integrity across various sectors. **UC1** focused on safeguarding nuclear facilities by addressing sophisticated **attacks on simulation codes** vital for their design. This involved a deep dive into protecting the integrity of input data during Uncertainty Quantification (UQ) processes, specifically within the URANIE platform. ^14^ The research aimed to prevent malicious alterations that could compromise the safety and operational efficiency of these critical infrastructures.

In the healthcare sector, two distinct use cases were investigated. **UC2** tackled the pervasive threat of **phishing email attacks designed to steal Electronic Health Record (EHR) data.** ^6, 7, 15^ The primary focus here was on attacks specifically targeting internal EHR systems, recognizing the severe implications of data breaches for patient privacy and trust. Simultaneously, **UC3** delved into the vulnerabilities of **Image Tampering in Healthcare**. This involved analyzing scenarios where adversaries attempted to alter AI models through data tampering during both training and inference phases, ^16, 17^ posing significant risks to diagnostic accuracy and treatment decisions.

The digital industry was represented by **UC4**, which analyzed the security requirements for **web bot detection.** ^18, 19^ This research concentrated on evaluating AI systems’ resilience against advanced, financially motivated cybercriminal bots, which can disrupt online services, manipulate data, and perpetrate fraud.

Finally, **UC5** investigated the **cyber-physical security of an AI-based rail detection system.** ^20, 21^ This system is crucial for Structural Health Monitoring (SHM) in critical infrastructure, and the research aimed to ensure its robust protection against cyber threats that could compromise its ability to detect structural anomalies and ensure safe rail operations.

To rigorously test and defend these systems, the project designed and developed an array of offensive and defensive tools.

In the realm of offensive capabilities, the partners successfully developed and deployed seven distinct prototypes, each targeting specific vulnerabilities and attack vectors across various use cases.

A sophisticated **simulation attack tool** was engineered to identify and exploit vulnerabilities inherent in the URANIE platform. ^14^ This tool achieved its objectives by manipulating input data streams and surreptitiously stealing or altering simulation code behavior, directly addressing the challenges outlined in Use Case 1 (UC1).

For social engineering scenarios, particularly within Use Case 2 (UC2), an **automated phishing engine** was meticulously designed and deployed. ^22^ This advanced system was capable of generating highly sophisticated, AI-driven phishing campaigns, ^22^ mimicking real-world threats and testing the resilience of human and technical defenses.

To simulate and counteract evasive digital attacks, a specialized **web bot detection and evasion tool** was developed. This tool primarily focused on advanced web scraping and scalping techniques, incorporating robust evasion support to bypass conventional detection mechanisms (UC2, UC4). ^18, 19^ Complementing this, **AI-enabled poisoning and Distributed Denial-of-Service (DDoS) evasion tactics** were implemented. These tactics specifically targeted and aimed to circumvent bot detection systems, demonstrating advanced methods for maintaining anonymity and persistence during malicious operations (UC4).

A **semi-automated penetration testing platform** was created to streamline reconnaissance and attack planning phases. This platform proved particularly effective when applied within the Structural Health Monitoring (SHM) use case, allowing for comprehensive vulnerability assessments and strategic attack simulations (UC5).

Furthermore, a dedicated **physical attack tool** was developed with a singular focus on creating and rigorously evaluating physical backdoor attacks. This tool specifically targeted the surface monitoring system, aiming to expose and exploit vulnerabilities at the hardware and physical access layers (UC5).

Finally, an **adversarial attack simulation tool** was employed to emulate various adversarial attack methods. This included techniques such as Projected Gradient Descent (PGD) and Trojaning, ^23, 24, 25^ with the primary goal of auditing the robustness and resilience of Artificial Neural Networks (ANNs). This was especially relevant for assessing their vulnerability to image tampering and other data manipulation tactics (UC3).

The defense mechanisms are composed of eight distinct AI detectors and defenders, each meticulously designed to bolster system responsiveness and robustness. A key focus in their development has been the integration of Explainable AI (XAI) principles, ^26^ ensuring that human expertise can be effectively incorporated and understood within the AI’s decision-making processes.

Among these crucial components are specialized tools for threat analysis and prevention:

• **Social Engineering Detection Tool:** This tool is specifically engineered to identify and flag attempts at social engineering, ^7, 22^ a tactic that manipulates individuals into divulging confidential information or performing actions beneficial to an attacker. It analyzes various communication channels and patterns for indicators of such sophisticated attacks.
• **Phishing Website Analysis Tool:** Complementing the social engineering detector, this tool focuses on analyzing malicious email content and URLs. It employs advanced techniques to detect fraudulent websites and links, ^15^ protecting users from falling victim to phishing schemes that aim to steal credentials or implant malware.

Furthermore, to combat automated threats and malicious network activity, the defense mechanisms include:

• **Bot Detection Systems:** These systems are designed for the comprehensive detection of malicious bot traffic. ^18, 19^ They employ sophisticated behavioral analysis, scrutinizing network interactions and user patterns to differentiate legitimate automated processes from malicious botnet activities, such as credential stuffing or denial-of-service attacks.
• **AI Module for Traffic Analysis:** Working in tandem with the bot detection systems, this AI module specializes in anomaly detection within network traffic. ^27^ By continuously monitoring and analyzing data flow, it can identify unusual patterns, sudden spikes, or deviations from established baselines that may indicate the presence of malicious bots or other cyber threats. This proactive approach allows for early detection and mitigation of potential attacks, enhancing the overall security posture of the system.

On the defensive front, eight autonomous AI detectors and defenders were developed. Protecting email users (UC2), a **social engineering detection tool** utilized Natural Language Processing (NLP) and machine learning to classify malicious messages and provided explanations using Explainable AI (XAI). This tool was often integrated with a **phishing website analysis tool**, which classified malicious phishing websites using hybrid URL and HTML analysis. For web bot defense (UC4), the project deployed a **general-purpose bot detector** combining heuristics and robust ML classification, alongside an **AI module for traffic analysis**, which learned legitimate traffic thresholds and incorporated human-in-the-loop (HITL) feedback. For nuclear simulation integrity (UC1), a **simulation defense tool** was designed as an anomaly detection module integrated directly into the platform, leveraging machine learning and HITL processes to validate input data. Addressing image tampering (UC3), a **hybrid defense system** was developed, which uniquely integrated XAI and a machine unlearning engine to detect and correct data poisoning ^16, 26, 28^ in medical AI models. For critical infrastructure (UC5), an **SHM defense tool** provided an Intrusion Detection System (IDS) based on Graph Neural Networks (GNN) ^27, 29^ to handle multi-modal data and detect anomalies on the central server. Finally, a **multidimensional data processing and intelligent framework** facilitated Cyber Situational Awareness (CSA) by aggregating and correlating security events (using IDMEFv2) ^30^ into a centralized Cyber Situational Picture (CSP) for operators (UC2, UC5), and a **cybersecurity simulation platform** served as a Decision Support System (DSS) utilizing Reinforcement Learning (RL) to train defender agents to suggest optimal countermeasures against complex threats like DDoS attacks (UC4, UC2).

A crucial early achievement was the definition of the tailored **KINAITICS Threat Matrix (KTM)**, which synthesizes knowledge from established frameworks, such as MITRE ATT&CK/ATLAS and the ENISA Threat Landscape Report, ^3, 5, 31^ to specifically align with the project’s unique focus on AI-enabled vulnerabilities. This framework provides direction across five distinct and complex use cases ^1, 4^ representing high-stakes sectors:

• Attacks on nuclear facility simulation codes (energy)
• Phishing attacks targeting electronic health records (EHR data)
• Image tampering in healthcare
• Web bot detection (digital industry)
• Cyber-physical security for rail detection systems (Structural Health Monitoring or SHM).

The comprehensive evaluation of the KINAITICS system has validated the efficacy of its integrated, AI-driven cybersecurity framework against sophisticated cyber-physical threats. This robust framework has demonstrated a significant advancement in securing critical infrastructures by proactively identifying and neutralizing complex attack vectors. The project’s core accomplishment lies in the successful development and operational integration of a multi-layered defense architecture, designed not only to enhance the resilience of these vital systems but also to provide continuous, adaptive protection in dynamic threat landscapes. This architecture leverages cutting-edge artificial intelligence and machine learning algorithms to detect anomalies, predict potential vulnerabilities, and respond swiftly to emerging threats, thereby ensuring the uninterrupted operation and integrity of critical infrastructure networks Figure 2.

The research was conducted in compliance with the principles stipulated in the Declaration of Helsinki. Informed consent for participation was obtained from all participants in Use Case 2 (EHR phishing defense evaluation). This part of the study was classified as a low-risk usability assessment involving a voluntary and anonymous survey (SUS and PSSUQ) following simulated phishing attacks. Consequently, participation in the survey after receiving comprehensive information about the simulated nature of the attacks was considered as implied verbal and informed consent. Written consent was deemed unnecessary due to the anonymity and low-risk nature of the data collection. All participants were adults, and no minors were involved.

## Results

This document details the rigorous evaluation of the KINAITICS system across five distinct use cases, showcasing its comprehensive capabilities in mitigating various cybersecurity threats. The comprehensive evaluation of the KINAITICS system has validated the efficacy of its integrated, AI-driven cybersecurity framework against sophisticated cyber-physical threats. The results for each use case demonstrator are detailed below.

### Use Case 1: Protecting nuclear simulation codes

The KINAITICS system demonstrated exceptional efficacy in safeguarding nuclear simulation codes from malicious attacks. ^14^ Its implemented defense mechanisms achieved a remarkably high success rate in detecting and blocking input-based attacks. Specifically, the system effectively identified and neutralized over 90% of malicious data points injected into the simulations. Beyond immediate threat mitigation, the integrated forensic and behavioral analysis tools played a crucial role in successfully confirming or, more importantly, preventing attempts at intellectual property exfiltration, thereby securing sensitive research and development. This robust performance highlights the system’s critical role in maintaining the integrity and confidentiality of vital nuclear research.

### Use Case 2: Defending Electronic Health Record (EHR) data from phishing attacks

In Use Case 2, the KINAITICS evaluation underscored the strength of its layered defense strategy against sophisticated multi-stage phishing attacks ^6, 7, 15^ targeting Electronic Health Record (EHR) data. The sequential application of specialized tools proved instrumental. The **social engineering detection tool** actively identified ^22^ and flagged deceptive tactics, while the **phishing website analysis tool** meticulously scrutinized suspicious URLs and content. Crucially, the integration of human-in-the-loop validation provided an indispensable layer of oversight, allowing human experts to confirm or override automated decisions, thus preventing threats that might otherwise evade single-point defenses. This comprehensive approach successfully measured the system’s ability to detect and block credential theft and data scraping attempts, confirming its effectiveness in protecting highly sensitive medical information and ensuring patient data privacy.

### Use Case 3: Countering adversarial attacks on diagnostic imaging models

The KINAITICS system’s performance in countering adversarial attacks on diagnostic imaging models ^28, 32^ was subjected to rigorous testing. Key findings definitively confirmed that AI-based tools, particularly the **hybrid defense system**, could effectively identify tampered images and models. The evaluation specifically focused on sophisticated attack vectors such as ^16, 17^ during the training phase to compromise the model’s integrity, and, ^23, 25^ which involve subtly altering input data to mislead the model during diagnosis. A critical finding was the incorporation of human radiologists into the decision process, ^26^ which proved to be an indispensable defense layer. Their expertise allowed for the verification of AI predictions and the identification of imperceptible perturbations that even advanced AI might miss, thereby enhancing diagnostic accuracy and patient safety.

### Use Case 4: AI-Based web bot detection

The evaluation of Use Case 4 validated the high performance of the KINAITICS AI-based systems designed for web bot detection. ^18, 19^ Demonstrator workshops provided a realistic proving ground, confirming the effectiveness of the **bot detection systems**, an **AI module for traffic analysis**, and a **cybersecurity simulation platform** against various forms of realistic bot traffic and adversarial evasion techniques. The system’s adaptive detection models, especially those trained via reinforcement learning, ^33, 34^ showcased a remarkable capability to distinguish between malicious and legitimate traffic in dynamic and evolving online environments. This was evidenced by an impressively low **false positive rate** of 0.795% and a **false negative rate** of 6.421% ^19^ in a supervised learning model, highlighting its precision in identifying and mitigating automated threats without unduly disrupting legitimate user activity.

### Use Case 5: Resilience against threats to Structural Health Monitoring (SHM) systems

Finally, in Use Case 5, the KINAITICS system’s resilience against threats to structural health monitoring (SHM) systems ^20^ was firmly established. The simulated scenarios successfully demonstrated the susceptibility of AI models within SHM systems to both **backdoor attacks,** ^24^ where hidden vulnerabilities are created for future exploitation, and **adversarial attacks**, which involve manipulating inputs to cause misclassifications. Countermeasures implemented, such as adversarial training (exposing models to adversarial examples during training to improve robustness) and prediction consistency checks (verifying the coherence of model outputs over time), proved highly effective in improving model robustness. This ensured accurate predictions of structural integrity even under malicious influence. Furthermore, the project successfully validated the use of ^27, 29^ for identifying infrastructure vulnerabilities and confirmed the system’s ability to detect and block attacks on the server and its connected nodes, thus ensuring the continuous and reliable operation of critical infrastructure.

The digital transformation of healthcare has led to widespread adoption of Electronic Health Record (EHR) systems ^6, 7^ to manage patient data. While this has streamlined patient care, it has also introduced new security vulnerabilities, making EHR data a prime target for cybercriminals. These attacks often employ **social engineering** and **phishing** ^15, 22^ to steal the credentials of healthcare professionals, which are then used to gain unauthorized access to sensitive patient information. The KINAITICS project addresses these threats through a multi-layered defense framework that includes tools for detecting and blocking sophisticated, multi-stage cyber attacks Table 1.

## Focus on attack to steal EHR data

KINAITICS Use Case 2, titled “Phishing email to steal EHR data,” is designed to evaluate how AI-based systems can detect attacks and non-conforming behavior during EHR consultation. This use case simulates a complete attack chain, from the initial social attack to the final data exfiltration, using a demonstration testbed that integrates various tools developed by consortium partners. The primary goal is to validate the effectiveness of these tools in protecting sensitive data and maintaining the integrity of IT services.

### The Anatomy of a Multi-Stage attack

The attack scenario is a four-step process, designed to mimic a realistic cyber-physical threat. ^6, 15^ The attack aims to steal credentials and exfiltrate sensitive data from the EHR system Figure 3.

The attack simulation unfolds as a meticulously planned, multi-stage process, designed to mimic real-world cyber threats that healthcare institutions face. Each step is carefully orchestrated, with corresponding defensive measures in place to highlight the interplay between offensive tactics and defensive strategies.

• **Step 1: Social Attack** 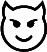  ○ The initial phase, a “Social Attack,” is essentially a sophisticated reconnaissance mission. It commences with the deployment of a **web bot detection and evasion tool.** ^18^ This tool is not just a simple scraper; it is designed to mimic legitimate user behavior while systematically harvesting publicly available information. It meticulously sifts through public-facing websites and various social media channels of a Partner hospital, aiming to gather critical intelligence such as employee email addresses, usernames, and even insights into organizational structure or recent events. The objective is to compile a rich dataset that will be instrumental in crafting a highly believable and targeted phishing campaign. The success of this initial reconnaissance significantly increases the probability of subsequent stages succeeding.
  ○ On the defensive front, an advanced **AI module for traffic analysis** is continuously at work. ^27^ This module utilizes machine learning algorithms to monitor incoming and outgoing web traffic in real-time. It is trained to identify subtle anomalies and patterns that are indicative of a social attack, such as unusual spikes in data requests from specific IP ranges, automated browsing behaviors that deviate from human interaction patterns, or attempts to access publicly available but sensitive information at an abnormal rate. By detecting these tell-tale signs, the AI aims to flag potential reconnaissance efforts before they can fully mature into a successful data harvesting operation Figure 4.
• **Step 2: Phishing Email** 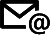  ○ Building upon the intelligence gathered in Step 1, the “Phishing Email” stage is launched by an **automated phishing engine**. This sophisticated tool leverages the harvested employee email addresses and usernames to craft highly personalized and convincing phishing emails. These emails are not generic spam; they are meticulously designed to appear legitimate, often mimicking internal communications, IT alerts, or urgent requests from known colleagues. Each email contains a malicious URL, carefully embedded to entice the recipient to click. The success of this stage hinges on the attacker’s ability to overcome user vigilance and existing email security measures.
  ○ The defense at this stage is multi-layered. Firstly, a **social engineering detection tool** meticulously analyzes ^22^ the incoming email’s headers and body. It scrutinizes various indicators, including sender authenticity, unusual phrasing, grammatical errors, and the presence of suspicious links or attachments. Based on this analysis, it calculates a “reliability score,” flagging emails that exhibit characteristics commonly associated with phishing attempts. Simultaneously, a dedicated **phishing website analysis tool** independently evaluates the embedded URL. This tool employs techniques such as sandbox execution, content analysis, and reputation checks to determine if the linked website is malicious or designed to steal credentials. An attack is considered successfully blocked if the recipient, through their training and awareness, recognizes the phishing attempt and avoids clicking the link. Alternatively, it is blocked if one of the automated defense tools successfully flags the email or its embedded URL as malicious, preventing the user from engaging with the threat Figure 5.
• **Step 3: Credential Stealing** 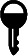

If the phishing email campaign proves successful and a user clicks on the malicious URL, the attack progresses to the “Credential Stealing” phase. ^6^ In this scenario, the user is seamlessly redirected to a cloned password-change website. This website is an exact replica of a legitimate internal portal or service, meticulously created by the same **automated phishing engine** used in Step 2. The uncanny resemblance is designed to lull the user into a false sense of security, making them believe they are interacting with a genuine system. When the user, unaware of the deception, proceeds to enter their credentials (username and password) on this fraudulent site, these sensitive details are immediately captured. These stolen credentials are then exfiltrated and sent directly to the **web bot detection and evasion tool** server, which now acts as a collection point for the illicitly obtained information. This critical step provides the attacker with the keys to potentially unlock various systems within the hospital network Figure 6.
• **Step 4: Data Scraping** 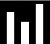

With the stolen credentials in hand, the attacker moves into the final and most damaging stage: “Data Scraping”. Using the compromised credentials, the attacker gains unauthorized access to the Electronic Health Record (EHR) system. ^6, 7^ This is the ultimate objective of the entire multi-stage attack. The attacker’s goal at this point is to exfiltrate sensitive and confidential patient data. This could involve targeting specific high-value information, such as the records of patients in a particular hospital ward, or focusing on highly sensitive data belonging to VIP patients. The success of this stage represents a significant data breach, potentially leading to severe consequences including privacy violations, reputational damage, and financial penalties for the affected healthcare institution. The attacker may employ automated tools to systematically download or copy large volumes of data, making the exfiltration process as efficient and covert as possible Figure 7.

### AI-Driven behavioral analysis for EHR attack detection and defense

The data scraping phase presents a unique challenge, as the attacker is using valid credentials. Traditional security measures, like signature-based firewalls, are ineffective against this type of insider threat. The KINAITICS defense relies on AI-powered behavioral analytics to detect the attacker’s activities, performed by tools like **bot detection systems** and a **multidimensional data processing and intelligent framework** during the data scraping stage.

### Role of bot detection systems and multidimensional data processing and intelligent framework

Tools such as **bot detection systems** ^18, 19^ and a **multidimensional data processing and intelligent framework** ingest action logs from the EHR system. They establish a baseline of normal user activity based on internal log features, including typical access patterns, data request volumes, and the types of records accessed by legitimate users. The AI models then compare real-time activity against this baseline. An attacker’s behavior—such as a high volume of data requests over a short period or attempts to access records outside of their normal scope—would trigger an alert as an anomaly. This is an example of an AI-driven anomaly detection model in a healthcare context.

• A **bot detection system** is designed to detect and mitigate malicious activities from bots. It uses a combination of techniques:
  ○ **Unsupervised learning**: An outlier detection module identifies unusual patterns ^18^ in HTTP request volumes over time intervals. It establishes a baseline of normal traffic from historical logs and dynamically adjusts thresholds.
  ○ **Supervised learning**: Boosted tree models classify requests as either benign or malicious based on features like request rate, user agent, and request patterns.

A core component of this system is a **multidimensional data processing and intelligent framework**, meticulously designed to analyze the forwarded EHR action logs. This sophisticated framework does not merely process data; it actively scrutinizes these logs for any anomalies or deviations from established policy. By employing advanced analytical techniques, it continuously looks for patterns that diverge from normal or expected usage within the EHR system. Upon the detection of any such anomaly or policy violation, the framework is engineered to immediately issue a warning, thereby providing an early alert to potential security breaches, misuse of data, or operational irregularities. This proactive approach is crucial for maintaining the integrity, security, and compliance of sensitive health information.

The Human-in-the-Loop Component

A key aspect of this defense strategy is the **human-in-the-loop** approach, ^26^ where security analysts can review and correct misclassifications. AI models, while powerful, can produce false positives or miss sophisticated attacks. A human operator is integrated into the decision-making process to validate the AI’s predictions and continuously improve the system. The **bot detection system**, for example, provides detailed insights and **counterfactual examples** to help analysts understand why a request was flagged as malicious. This feedback is crucial for retraining and improving the models over time, ensuring they can adapt to new and evolving threats.

• **Validation and Feedback Loop**: When an anomaly is detected, the system generates an alert. A human security analyst reviews this alert and, using their expertise, confirms if the activity is a real threat or a false positive. This human feedback is used to retrain the AI model, reinforcing correct classifications and correcting errors. This iterative process is crucial for making the models more accurate and robust over time.
• **Explainable AI (XAI)**: To assist human operators, the system leverages XAI. ^26^ For example, the **bot detection system** can generate **counterfactual examples** to explain why a request was classified as malicious. An explanation might show that if the request rate were slightly lower, or the user agent was recognized as legitimate, the classification would have been different. This transparency reduces the cognitive load on analysts, allowing for faster, more accurate decision-making Figure 8.

### Scenarios and outcomes

In the event of a successful data scraping attack, the goal is to detect and block the malicious activity before data exfiltration is complete. The scenarios test various outcomes based on whether the tools and the human user successfully identify the attack.

• If the **bot detection system** detects an anomaly, it signals an account blocker, effectively blocking the attack.
• If the **multidimensional data processing and intelligent framework** detects an anomaly, it shows a warning to the operator.
• An attack is considered successful if all defense mechanisms fail to detect the scraping activity.

### Evaluation and validation

The effectiveness of this use case is measured using two key performance indicators (KPIs): **Percentage of Attacks Detected** and **Percentage of Attacks Blocked**. User feedback, collected through questionnaires like the System Usability Scale (SUS) and the Post-Study System Usability Questionnaire (PSSUQ), ^35^ also plays a vital role in evaluating the system’s usability and usefulness. The results of the evaluation for this use case demonstrated a robust, multi-layered defense strategy against phishing and data scraping attacks on EHR systems.

User feedback was collected via the PSSUQ questionnaire after participants interacted with the related tools during simulated phishing attacks, with a focus on user interaction at various stages, including the human user’s ability to recognize a phishing email. The feedback highlighted the value of real-time logging and alerts in supporting human validation, particularly when automated system detection failed.

Sample Size: SUS: 7 responses, PSSUQ: 7 responses. Table 2, Figure 9.

## Discussion

Use Case 2 of the KINAITICS project successfully demonstrates a comprehensive, multi-stage defense strategy against EHR data theft. By combining AI-based tools like **bot detection systems** and a **multidimensional data processing and intelligent framework** with a human-in-the-loop validation process, ^18, 26^ the system can effectively detect and block a full chain of attack, from social engineering to data scraping. This layered approach not only protects sensitive patient information but also provides a framework for continuous improvement of the defense mechanisms, which is essential for adapting to the ever-evolving threat landscape Figure 10.

The comprehensive evaluation of Use Case 2 within the KINAITICS project illustrates the efficacy of a multi-layered, AI-driven defense architecture for protecting Electronic Health Record (EHR) systems against sophisticated credential-stealing and data-exfiltration attacks. By orchestrating a sequence of automated tools—ranging from social engineering detection and phishing website analysis to bot detection systems and multidimensional data processing frameworks—alongside a human-in-the-loop validation process, the system successfully intercepts each stage of a realistic, multi-step attack chain.

Key takeaways include:

• **Holistic Threat Coverage:** The integration of natural language processing for email analysis, machine-learning classifiers for URL and traffic monitoring, and anomaly-detection modules ^18, 22^ at the application layer ensures that both overt and subtle attack vectors are identified and mitigated before data can be exfiltrated.
• **Human-in-the-Loop Feedback Loop:** Incorporating security analysts to review alerts and counterfactual explanations enhances model accuracy over time. This iterative retraining process reduces false positives and adapts the system to emerging attack patterns, ensuring robust, context-aware protection.
• **Explainable AI (XAI) Transparency:** By providing counterfactual examples and clear reasoning for each alert, the framework lowers the cognitive burden on analysts, ^26^ speeds up incident response, and fosters greater trust in automated defenses.
• **Continuous Improvement and Adaptability:** Real-time logging of attack attempts and user feedback via SUS and PSSUQ assessments (Overall SUS 68.2; PSSUQ 2.00) highlight usability strengths and areas for refinement. The defense funnel design—with explicit checkpoints at reconnaissance, phishing, credential capture, and data scraping ^6, 26^—serves as both an operational blueprint and a metrics-driven dashboard for ongoing optimization.

**Future Directions:** To maintain resilience against rapidly evolving threats, future enhancements will explore:

• Advanced graph-based anomaly detection ^27^ to capture complex multi-user correlations.
• Federated learning approaches for cross-institutional intelligence sharing without compromising privacy.
• Autonomous remediation capabilities that can isolate suspect sessions in real time.
• Expanded human-machine collaboration tools, such as interactive XAI dashboards and guided decision-support workflows.

## Conclusion

Ultimately, the KINAITICS project successfully demonstrates a comprehensive, multi-stage defense strategy against advanced cyber-physical threats across five high-stakes sectors. The evaluation, particularly in Use Case 2, validates a scalable, adaptive defense framework capable of safeguarding patient privacy and institutional integrity in digital healthcare environments. By blending cutting-edge AI models with human expertise and continuous feedback, the KINAITICS approach sets a new standard for proactive, intelligence-driven cybersecurity in critical infrastructures.

## Declaration

Generative Artificial Intelligence (AI) tools were utilized for the preparation of Figures (specifically Napkin, available at https://www.napkin.ai/) and for the correction of English grammar and phrasing within the manuscript (using Gemini 2.5, and 3.0).

## Ethics and consent

All research was conducted in compliance with the principles stipulated in the Declaration of Helsinki ( https://www.wma.net/policies-post/wma-declaration-of-helsinki/).

## Ethical oversight

Formal ethical approval from an Institutional Review Board (IRB) or Research Ethics Committee (REC) was not required for this study. This determination was made because the activity was classified as a low-risk usability assessment involving only voluntary and anonymous surveys, and did not constitute a clinical protocol, experimentation on human or animal subjects, or collection of sensitive data as defined by the GDPR. Ethical oversight was instead governed by the project’s official Data Management Plan (DMP), which details the data processed (anonymized data from experts) and its management modalities, as officially approved by the European Project Officer and reviewers.

## Participants and consent

The study participants were adult cybersecurity experts, who voluntarily adhered to the demonstrator workshops for the Use Case 2 evaluation (EHR phishing defense). No minors were involved.

Informed consent for participation and anonymized and aggregated data publication was reported and obtained in the questionnaires. All participants received comprehensive information about the simulated nature of the phishing attacks. The activities involving participants within the EU KINAITICS project (Grant Agreement 101070176) were reviewed and granted an exemption from full clinical IRB review by the Institutional Review Board of the Fondazione Toscana Gabriele Monasterio.

## Data Availability

The relevant data can be accessed via the Zenodo repository at the following DOI: https://doi.org/10.5281/zenodo.18302400. ^36^ Data are available under the terms of the Creative Commons Attribution 4.0 International license (CC-BY 4.0).

## References

[1] KINAITICS Consortium: KINAITICS: Cyber-kinetic attacks using Artificial Intelligence. 2024. Reference Source

[2] European Commission: Cyber-kinetic attacks using Artificial Intelligence. *CORDIS EU Research Results* 2022. Reference Source

[3] ENISA: *ENISA Threat Landscape 2025.* European Union Agency for Cybersecurity;Oct. 2025. Reference Source

[4] Kafali E : *Crafting a Tailored Threat Matrix for KINAITICS.* CERTH;Jan. 2024. Reference Source

[5] MITRE Corporation: MITRE ATLAS: Adversarial Threat Landscape for Artificial-Intelligence Systems. 2024. Reference Source

[6] HIPAA Journal: Healthcare Data Breaches Due to Phishing. Sep. 2024. Reference Source

[7] Yeo LH Banerjee A : Human Factors in Electronic Health Records Cybersecurity Breach: An Exploratory Analysis. *J. Med. Internet Res.* Mar. 2022. PMC9123525 35692854

[8] Marcheschi P Mazzarisi A Dalmiani S : HL7 clinical document architecture to share cardiological images and structured data in next generation infrastructure. *Comput. Cardiol.* 2004;2004:617–620. 10.1109/CIC.2004.1443014

[9] Dalmiani S Morales MA Mazzarisi A : EMR standards in cardiological outpatient management. *Comput. Cardiol.* Sep. 2004;2004:613–616. 10.1109/CIC.2004.1443013

[10] Marcheschi P Mazzarisi A Dalmiani S : New standards for cardiology report and data communication: an experience with HL7 CDA release 2 and EbXML. *Comput. Cardiol.* Sep. 2005;2005:383–386. 10.1109/CIC.2005.1588117

[11] Marcheschi P Mazzarisi A Marraccini P : ‘Effective teleconsulting and teleconferencing using standard DICOM protocol and push technology on low bandwidth connection’, in. *Comput. Cardiol.* Sep. 2003;2003:686–688. 10.1109/CIC.2003.1291248

[12] Marcheschi P Mazzarisi A Dalmiani S : ‘ECG standards for the interoperability in patient electronic health records in Italy’, in. *Comput. Cardiol.* Sep. 2006;2006:549–552. Reference Source

[13] Marcheschi P Positano V Ferdeghini EM : An open source based application for integration and sharing of multi-modal cardiac image data in a heterogeneous environment. *Comput. Cardiol.* Sep. 2003;2003:367–370. 10.1109/CIC.2003.1291168

[14] Blanchard J-B Damblin G Martinez J-M : The Uranie platform: an Open-source software for optimisation, meta-modelling and uncertainty analysis. *ArXiv Prepr. ArXiv180310656.* Mar. 2018. Reference Source

[15] Bluesight: Phishing Attacks: A Hacker’s Gateway to Patient Health Records. Mar. 2025. Reference Source

[16] Pawelczyk M Di JZ : Machine Unlearning Fails to Remove Data Poisoning Attacks. *ArXiv Prepr. ArXiv240617216.* Jun. 2024. Reference Source

[17] Nasim MAA Biswas P : Securing the Diagnosis of Medical Imaging: An In-depth Analysis of AI-Resistant Attacks. *ArXiv Prepr. ArXiv240800348.* Jul. 2024. Reference Source

[18] Bunny.net: Bot Detection and Mitigation Techniques. 2024. Reference Source

[19] Radware: Role of Machine Learning in Thwarting Automated Bot Attacks. Oct. 2025. Reference Source

[20] Armijo A Martinez J : Integration of Railway Bridge Structural Health Monitoring into Digital Twin. *Sensors.* Mar. 2024;24. 10.3390/s24072115 38610327 PMC11013990

[21] Encardio: AI in Civil Infrastructure and Structural Health Monitoring. 2024. Reference Source

[22] Mittal A Chowdhury T Sivaraman R : Phishing Detection Using Natural Language Processing and Machine Learning. *SMU Data Sci. Rev.* 2022;5(2).

[23] Geisler S Wollschläger T : Attacking Large Language Models with Projected Gradient Descent. *ArXiv Prepr. ArXiv240209154.* Feb. 2024. Reference Source

[24] Gu X Fields G : Trojan Cleansing with Neural Collapse. *ArXiv Prepr. ArXiv241112914.* Dec. 2022. Reference Source

[25] Kaggle: Understanding PGD Attacks in Deep Learning. Jan. 2024. Reference Source

[26] Alkhanbouli R : The role of explainable artificial intelligence in disease prediction. *BMC Med. Inform. Decis. Mak.* Mar. 2025;25:110. 10.1186/s12911-025-02944-6 40038704 PMC11877768

[27] Ahanger AS : Advanced intrusion detection in internet of things using Graph Neural Networks. *Sci. Rep.* Mar. 2025;15:9831. 10.1038/s41598-025-94624-8 40119119 PMC11928630

[28] Dietrich N Wu S : Adversarial artificial intelligence in radiology: Attacks, defenses, and implications. *Diagn. Interv. Imaging.* May 2025;106:375–384. 10.1016/j.diii.2025.05.006 40404555

[29] Xu R Wu G : Applying Self-supervised Learning to Network Intrusion Detection for Network Flows with Graph Neural Network. *ArXiv Prepr. ArXiv240301501.* Mar. 2024;248:110495. 10.1016/j.comnet.2024.110495 Reference Source

[30] IDMEFv2 Task Force: The Incident Detection Message Exchange Format version 2 (IDMEFv2). May 2023. Reference Source

[31] ENISA: *ENISA AI Threat Landscape Report.* European Union Agency for Cybersecurity;Dec. 2020. Reference Source

[32] Tsai MJ Tao YH : Adversarial Attacks on Medical Image Classification. *Sci. Rep.* Aug. 2023. 10.1038/s41598-023-40813-y PMC10487122 37686504

[33] Landolt CR Würsch C : Multi-Agent Reinforcement Learning in Cybersecurity: From Fundamentals to Applications. *ArXiv Prepr. ArXiv250519837.* May 2025. Reference Source

[34] Finistrella S Mariani S Zambonelli F : Multi-agent Reinforcement Learning for Cybersecurity: Approaches and Challenges. *Workshop From Objects to Agents.* 2024;26:200495. 10.1016/j.iswa.2025.200495

[35] W. T: PSSUQ (Post-Study System Usability Questionnaire). *UIUX Trend.* Accessed: Dec. 10, 2025. Reference Source

[36] Marcheschi P Pisani M Dalmiani S : Kinaitics SUS and PSSUQ. Jan. 2026. 10.5281/zenodo.18302400 PMC13324364 42404426

