# Supplementary material for: From Simulation to Healthcare: KINAITICS' AI Framework for Cyber-Physical Security
Source: Open Res Eur. 2026 May 15;6:170. [Version 1] doi: 10.12688/openreseurope.21717.1 (PMC13324364; doi:10.12688/openreseurope.21717.1)
Supplement: Peer review history

# Reviewer report: Reviewer response for version 1

**DOI:** 10.21956/openreseurope.23495.r75017

**Published:** 2026-07-01

**Authors:** Brian Kee Mun Wong

**Affiliations:** 1 Swinburne University of Technology Sarawak Campus, Kuching, Malaysia

This paper presents the KINAITICS framework, which addresses emerging cyber-kinetic threats enabled by artificial intelligence (AI) in cyber-physical systems (CPS). The study is timely, aiming to bridge cybersecurity, AI robustness, simulation environments, and operational healthcare applications through a unified threat-modelling and defence framework. This is highly relevant given the increasing deployment of AI-driven control and perception systems in critical infrastructures and healthcare environments.

 The key contribution is the KINAITICS Threat Matrix (KTM) and its application across five use cases (UC1–UC5) (refer to Table 1, Figure 2). The interdisciplinary scope, covering nuclear simulation security, EHR phishing defence, medical image tampering, web-bot detection, and rail structural health monitoring are indeed well combined with attention to human-in-the-loop and explainable AI (XAI) elements.

 However, the manuscript currently reads more as a project overview than a comprehensive scientific report. The evidentiary base supporting its core claims can be further enhanced, while several methodological and reporting issues can be better addressed.

**Suggestions**

 (1) Use Case 2 (EHR phishing defence) receives a detailed methodological walkthrough (Figures 3–7, Table 2) and is the only use case with quantitative usability data (SUS = 68.2, PSSUQ = 2.00; Figure 9, Table 2). However, UC1, UC3, UC4 and UC5 are each reported in a single paragraph with headline figures (e.g. “over 90%” detection in UC1, and false positive/false negative rates of 0.795%/6.421% in UC4) with no further description. Hence, suggest each use case to report comparable detail (e.g. dataset/scenario description, number of trials, statistical variability, specific detection/blocking KPIs used).

 (2) The SUS/PSSUQ evaluation in UC2 is based on only seven participants (refer to Table 2). While the resulting SUS score (68.2) is described in the paper as “good usability,” this conclusion is not statistically supported by a small sample size. Suggest that the authors explicitly acknowledge this as a limitation, report confidence intervals, and avoid descriptive labels (“good usability”) that imply a level of certainty the sample does not support.

 (3) Given that the KTM’s novelty lies in addressing threats “unique to AI-integrated CPS,” the authors may clarify, in the Introduction, the operating definitions of the core terms such as cyber-attack, cyber-physical attack, cyber-kinetic attack, and AI-enabled attack, instead of being used interchangeably. Besides, the authors may also clarify how the relationship between cyber events, physical consequences, and AI-specific vulnerabilities is conceptualised.

 (4) The KTM is described as synthesising MITRE ATT&CK/ATLAS and the ENISA Threat Landscape Report, but the paper does not explain what the KTM adds beyond these existing frameworks, nor benchmark it against alternative approaches (e.g. STRIDE, digital-twin-based security simulations, or other AI-security taxonomies). Hence, a dedicated comparison section, situating KINAITICS within the AI security, CPS security, healthcare cybersecurity, and adversarial-ML literature, would substantially strengthen the claimed novelty.

 (5) One of KINAITICS’ potentially distinctive contributions is incorporating human behaviour and uncertainty into cyber-defence modelling (the human-in-the-loop cycle in Figure 8). Perhaps the authors may provide more detail on the underlying human-behaviour models, user-response simulations, cognitive assumptions, or how these models were validated. This contribution can be significant for the study, hence worth some expansion thoughts.

 (6) The ethics statement should be consistent from one section to another. For example, the Ethics and Consent section first states that “formal ethical approval from an Institutional Review Board (IRB) or Research Ethics Committee (REC) was not required,” and shortly after states that the activities “were reviewed and granted an exemption from full clinical IRB review by the Institutional Review Board of the Fondazione Toscana Gabriele Monasterio.” These two statements appear contradictory and should be reconciled and clarified.

 (7) Suggest the authors provide a consolidated acronym/abbreviation table (CPS, KTM, XAI, HITL, SHM, IDS, GNN, CSA, CSP, DSS, RL, UQ, PGD, etc.) given the density of project-specific terminology.

Is the case presented with sufficient detail to be useful for teaching or other practitioners?

Yes

Is the work clearly and accurately presented and does it cite the current literature?

Yes

If applicable, is the statistical analysis and its interpretation appropriate?

Yes

Are all the source data underlying the results available to ensure full reproducibility?

Yes

Are the conclusions drawn adequately supported by the results?

Yes

Is the background of the case’s history and progression described in sufficient detail?

Yes

Reviewer Expertise:

Healthcare Travel Tech, Tourism, Marketing, Management

I confirm that I have read this submission and believe that I have an appropriate level of expertise to confirm that it is of an acceptable scientific standard.

# Reviewer report: Reviewer response for version 1

**DOI:** 10.21956/openreseurope.23495.r75011

**Published:** 2026-06-19

**Authors:** R Renuga Devi

**Affiliations:** 1 SRM Institute of Science and Technology, Chennai, India

This paper presents a comprehensive and timely investigation into AI-enabled cyber-kinetic threats targeting Cyber-Physical Systems (CPS). The proposed KINAITICS Threat Matrix (KTM) offers a structured framework for identifying and assessing vulnerabilities across critical sectors, including healthcare, nuclear facilities, and transportation systems. The integration of offensive and defensive AI tools, coupled with Explainable AI and human-in-the-loop mechanisms, significantly strengthens the practical relevance of the work. Extensive evaluation across multiple use cases demonstrates the effectiveness of the proposed framework in enhancing cyber resilience. Overall, the study provides a valuable contribution to the field of AI-driven cybersecurity and critical infrastructure protection. 

 Suggestions: 

 The machine learning and deep learning models used in each defense tool should be explicitly specified, including model architecture, training datasets, feature selection methods, and hyperparameter settings. 

 Performance evaluation metrics should be reported more comprehensively. Along with detection rates, metrics such as Precision, Recall, F1-Score, ROC-AUC, and computational overhead should be included. 

 The paper reports successful attack detection rates (>90%) but lacks statistical significance analysis and confidence intervals. Including these would improve the scientific rigor of the results.

Is the case presented with sufficient detail to be useful for teaching or other practitioners?

Yes

Is the work clearly and accurately presented and does it cite the current literature?

Yes

If applicable, is the statistical analysis and its interpretation appropriate?

Yes

Are all the source data underlying the results available to ensure full reproducibility?

Yes

Are the conclusions drawn adequately supported by the results?

Yes

Is the background of the case’s history and progression described in sufficient detail?

Yes

Reviewer Expertise:

NA

I confirm that I have read this submission and believe that I have an appropriate level of expertise to confirm that it is of an acceptable scientific standard.
